# Effects of dietary supplementation with quercetagetin on nutrient digestibility, intestinal morphology, immunity, and antioxidant capacity of broilers

**DOI:** 10.3389/fvets.2022.1060140

**Published:** 2022-12-23

**Authors:** Fengyang Wu, Haonan Wang, Shuo Li, Zhonghua Wei, Shuaijuan Han, Baojiang Chen

**Affiliations:** ^1^College of Animal Science and Technology, Hebei Agricultural University, Baoding, China; ^2^College of Food Science and Technology, Hebei Agricultural University, Baoding, China; ^3^College of Animal Science, Guizhou University, Guiyang, China; ^4^Broiler Test Station, Institute of Animal Husbandry and Veterinary Medicine of Hebei Province, Baoding, China

**Keywords:** quercetagetin, broiler, nutrient digestibility, intestinal morphology, immunity, antioxidant capacity

## Abstract

Quercetagetin (QG) is gaining increased attention as a potential alternative to in-feed antioxidants due to its antioxidant activity. This experiment was conducted to investigate the effects of dietary supplementation with QG on nutrient digestibility, intestinal morphology, immunity, and antioxidant capacity of broilers. Four hundred 1-day-old Ross 308 broilers were randomly assigned into 4 groups with 10 replicates in each group and 10 broilers in each replicate. The four dietary treatments included the basal diet supplemented with 0, 3.2, 4.8, or 6.4 mg/kg QG. The results showed that dietary supplementation with QG significantly promoted the broilers' apparent digestibility of phosphorus (*P* < 0.05), increased the villus height in jejunum and ileum, and reduced the crypt depth in jejunum and ileum, which significantly increased the ratio of villus height to crypt depth in the jejunum and ileum (*P* < 0.05). The dietary supplementation with QG also significantly enhanced the immunoglobulin G (IgG) and complement 4 (C4) levels in the blood (*P* < 0.05), the activity of total antioxidant capacity (T-AOC) in serum, jejunum mucosa, and ileum mucosa, the activity of superoxide dismutase (SOD) in the serum and liver (*P* < 0.05), and significantly up-regulated the kelch-like ECH-associated protein 1 (*Keap1*), nuclear factor E2 related factor 2 (*Nrf2*), heme oxygenase-1 (*HO-1*), NAD(P)H: quinone oxidoreductase 1 (*NQO-1*), glutathione peroxidase (*GSH-Px*) and superoxide dismutase 1 (*SOD1*) mRNA expression levels in the jejunum mucosa, ileum mucosa, and liver tissues of broilers. Therefore, supplementing broilers' diets with QG can enhance the apparent digestibility of phosphorus, improve the structure and morphology of jejunum and ileum, promote immunity, and increase the activity of antioxidant enzymes and the antioxidantive capacity through the Nrf2/antioxidant response element (ARE) signaling pathway mediated by Keap1.

## 1. Introduction

Normally, fewer free radicals are produced of poultry, which can be timely eliminated by the antioxidant system to prevent oxidative stress and injury (1). However, with the increase in poultry production scale and intensity, various factors, such as excessive feeding, lower feeding management, unstable feed quality (excessive metal element content, mycotoxin pollution, and lipid oxidation), and pathogen infestation increase the free radical production in poultry through the respiratory burst mechanism, which exceeds the scavenging capacity of the antioxidant system, triggering a free radical chain reaction, which leads to oxidative stress, and damage (2–7). In chicks, oxidative stress also reduces disease resistance, survival rate, feed return, and production performance and causes maldevelopment. In adult chickens, oxidative stress reduces immunity, lowers product quality, and decreases production performance (8, 9).

Enhancing the anti-stress potential and reducing the negative effects of oxidative stress on poultry production is the key to transforming, upgrading, and developing a green and high-quality poultry breeding industry. Quercetagetin (C_15_H_10_O_8_, QG) is a flavonol compound chemically identified as 3,3,4,5,6,7-hexahydroxyflavone extracted from marigold (*Tagetes erecta* L.) (10). QG is a safe, efficient, and economical natural antioxidant (11), with strong scavenging activities against hydroxyl radical (OH·), 2,2 -biphenyl-1-picrylhydrazino (DPPH), and 2,2′- diazabibis (3-ethylbenzothiazolin-6-sulfonic acid) diammonium salt (ABTS) (12).

QG has greater development potential and research value in alleviating oxidative stress and improving the antioxidant capacity of poultry. However, there are few reports on the influence of QG on broiler production. Therefore, this study evaluated the effects of different QG dosages as dietary supplements on nutrient digestibility, intestinal morphology, immunity, and antioxidant capacity of broilers. The findings in this study will provide a reference for the application of QG in broiler production.

## 2. Materials and methods

### 2.1. Experimental diet

Quercetagetin (>80% purity) was purchased from Chenguang Biotech Group Co., Ltd. (Handan, China). The basal diet was formulated according to National Research Council (NRC) (13). The composition and nutritional levels of the basal diet is shown in Table 1.

**Table 1:** Composition and nutrient levels of the basal diet (air-dry basis, %).

**Item**	**Trial period**	
	**1–21 d**	**22–42 d**
**Ingredients**		
Corn	60.00	64.37
Soybean meal	28.37	25.20
Fishmeal	5.00	2.00
Vegetable oil	3.00	5.00
CaHPO_4_	1.40	1.20
Limestone	1.20	1.30
C_5_H_14_CINO	0.10	0.10
Premix^a^	0.18	0.18
Lysine	0.35	0.25
Methionine	0.15	0.10
NaCl	0.25	0.30
Total	100.00	100.00
**Nutrient levels** ^b^		
Metabolic energy (MJ/kg)	13.25	13.74
Crude protein	21.35	20.02
Calcium	1.00	0.95
Available phosphorus	0.48	0.44
Total phosphorus	0.68	0.65
Lysine	1.16	1.03
Methionine	0.47	0.40

a The premix provided the following per kg of diets: vitamin A, 10,000 IU; vitamin D, 34,000 IU; vitamin E, 28 mg; vitamin K3, 3 mg; vitamin B1, 4 mg; vitamin B2, 10 mg; vitamin B6, 6 mg; vitamin B12, 0.08 mg; niacin, 1,000 mg; pantothenic acid, 18 mg; pyridoxine, 8 mg; folic acid, 1 mg; biotin, 0.3 mg; C_5_H_14_ClNO, 600 mg; Fe, 60 mg; Cu, 10 mg; Zn, 80 mg; Mn, 90 mg; I, 1 mg; Se, 0.3 mg.

b Metabolic energy, Crude protein, Calcium and Total phosphorus were calculated value, while the others were measured values.

### 2.2. Experimental design, animal, and management

Four hundred 1-day-old Ross 308 broilers were randomly assigned into 4 groups (three experimental groups and control) with 10 replicates in each group and 10 broilers in each replicate. The control group was fed with the basal diet, while the experimental groups were fed on the basal diet supplemented with 3.2, 4.8, and 6.4 mg/kg QG (calculated based on a 100% purity). The broilers were reared for 42 d.

The experimental protocols were approved by the Animal Care and Use Committee of Hebei Agriculture University (Baoding, China).

All animal experiments complied with the ARRIVE guidelines were carried out in accordance with the U.K. Animals (Scientific Procedures) Act, 1986 and associated guidelines, EU Directive 2010/63/EU for animal experiments.

### 2.3. Determination indexes and methods

#### 2.3.1. Nutrient digestibility

The nutrient digestibility of broilers in each group was measured using the endogenous indicator method (acid-insoluble ash). Fresh feces were collected from each group five days to the end of the experimental period. The feces were weighed and assigned to two fecal samples. One sample was mixed with 10% hydrochloric acid to determine the level of nitrogen fixation and crude protein content. The second sample was used to detect the contents of other nutrients. All fecal samples were dried, crushed, and placed into a sample bottle for subsequent testing. The chemical composition of the ingredients was determined as proposed by AOAC (14). The basal diet nutrient composition in each group was also determined. The apparent digestibility of nutrients in each group was determined as follows:


Nutrient digestibility % = 100−100(b×c)÷(a×d)× 100.

Where a is the content of X nutrient in feed; b is the content of X nutrient in fecal samples; c is the content of acid-insoluble ash in feed; d is the content of acid-insoluble ash in fecal samples.

#### 2.3.2. Indicators in serum and tissue

At the end of the experimental period, the broilers were fasted for 12 h; then, one chicken was randomly selected from each replicate for blood collection (10 mL) from the wing vein. The blood samples were collected in vacutainers and centrifuged at 3,000 ×g at 4°C for 10 min to separate the serum. Serum samples were stored at −20°C until further analysis. In addition, one chicken/replicate was randomly selected and euthanized by cervical dislocation. The jejunum, ileum, and liver samples were collected using surgical instruments that had been rigorously sterilized, then cryopreserved at −80°C for testing. The levels/activities of immunoglobulin A (IgA), immunoglobulin G (IgG), immunoglobulin M (IgM), complement 3 (C3), complement 4 (C4), total antioxidant capacity (T-AOC), glutathione peroxidase (GSH-Px), superoxide dismutase (SOD), and malondialdehyde (MDA) in the blood and tissues were determined to use ELISA kits [Nanjing Jiancheng Bioengineering Institute (Nanjing, China)] according to the manufacturer's instructions.

#### 2.3.3. The jejunum and ileum tissues morphology

The jejunum and ileum tissues were picked under aseptic conditions and fixed using a 10% neutral formaldehyde fixing solution. The fixed tissues were dehydrated using a full-automatic dehydrator (TSJ-II, Zhongshan, Changzhou, China). After embedding and slicing, the following operations were performed: the fixed tissues were subjected to slice dewaxing. After that, it was put in hematoxylin stain for 15 min and rinsed with tap water for 2 min. Thereafter, hydrochloric acid alcohol differentiation was done for 10 s, rinsed with tap water for 2 min, and put in warm water at 50°C until the solution turned blue. It was then rinsed with tap water for 2 min, and put in 85% alcohol for 4 min. The tissues were then stained with eosin for 4 min, washed with distilled water for 5 s. The tissues were then dehydrated using gradient alcohol, made transparent using xylene, and sealed with neutral gum. The trinocular biological microscope camera system (BA200 digital, motic, Xiamen, China) was used for slice observation and image acquisition. All tissues of each section were observed using a microscopic camera system at ×40, and the tissue images were taken at ×100 and ×400.

#### 2.3.4. Relative gene expression in the tissues

The relative content of mRNA of tissue samples were detected by quantitative real-time PCR (qRT-PCR). The specific primers were designed using primer 6.0 software based on the gene sequences of kelch-like ECH-associated protein 1 (*Keap1*), nuclear factor E2 related factor 2 (*Nrf2*), heme oxygenase-1 (*HO-1*), NAD(P)H: quinone oxidoreductase 1(*NQO-1*), *GSH-pX*, superoxide dismutase 1 (*SOD1*), glyceraldehyde-3-phosphate dehydrogenase (*GAPDH*) in the GenBank and were synthesized by Bioengineering Co., Ltd. (Shanghai, China) (Table 2).

**Table 2:** Sequence of primers for real-time PCR.

**Target gene**	**Primer sequence (5^′^to 3^′^)**	**Product size (bp)**	**GenBank accession no**.
*Keap1*	Forward: CCAACTTCGCCGAGCAGA	120	XM_010728179.2
	Reverse: GCTGGCAGTGGGACAGGTT		
*Nrf2*	Forward: CACCAAAGAAAGACCCTCCT	197	XM_015289381.3
	Reverse: GAACTGCTCCTTCGACATCA		
*HO-1*	Forward: CCGCTATTTGGGAGACCT	166	NM_205344.1
	Reverse: CTCAAGGGCATTCATTCG		
*NQO-1*	Forward: TCTCTGACCTCTACGCCAT	93	NM_001277621.1
	Reverse: TCTCGTAGACAAAGCACTCGG		
*GSH-Px*	Forward: GATGAGATCCTGAGAGTGGTGGAC	116	NM_000581.4
	Reverse: TCATCAGGTAAGGTGGGCACAA		
*SOD1*	Forward: AGGGAGGAGTGGCAGAAGT	163	NM_205064.1
	Reverse: GCTAAACGAGGTCCAGCAT		
*GAPDH*	Forward: GGCTGCTAAGGCTGTGGG	136	NM_204305.1
	Reverse: ATCATCATACTTGGCTGGTTTC		

Keap1, kelch-like ECH-associated protein 1; Nrf2, nuclear factor E2 related factor 2; HO-1, heme oxygenase-1; NQO-1, NAD(P)H: quinone oxidoreductase 1; GSH-Px, glutathione peroxidase; SOD1, superoxide dismutase 1.

The total RNA was extracted using 50–100 mg of the jejunal, ileal and hepatic samples, respectively, following the Trizol reagent according to the manufacture's instruction (Invitrogen, Carlsbad, USA). The extracted RNA concentration was detected using an RNA concentration meter (Nanodrop Lite, Thermo Fisher Scientific, Massachusetts, USA). A total of 1 μg RNA were used for cDNA synthesis with the kit of HiScript III RT SuperMix for qPCR (+gDNA wiper) (Number: R323-01, Vazyme, Nanjing, China) based on the manufacture's instruction. The qRT-PCR was performed with CFX96 touch real-time PCR detection system (Bio-Rad, Hercules, CA, USA) using the primers displayed in Table 2. GAPDH was selected as internal control to compare the amplification efficacy. The ChamQ Universal SYBR qPCR Master Mix (Number: Q711-02, Vazyme, Nanjing, China) was used in qRT-PCR. A total of 20 μL qRT-PCR mixture consisted of 10 μL SYBR Green Master Mix, 0.8 μL of forward and reverse primers mix (stock concentration of 10 μmol/L), 2 μL (200 ng) template cDNA and 7.2 μL DNase/RNase Free H_2_O. The heat-cycling conditions of qRT-PCR: 95°C for 5 min, followed by 40 cycles at 95°C for 10 s, 60°C for 30 s of melt curve analysis. The experiment was repeated three times and the target gene expression was calculated by the 2^−ΔΔCt^ method.

### 2.4. Statistical analysis

The statistical data analysis was done using Excel 2016 and SPSS 20.0 software. One-way analysis of variance (ANOVA) was used to test the significant differences between each group data, while the Duncan method was used for multiple comparisons. A *p*-value < 0.05 (*P* < 0.05) showed a significant difference between the groups.

## 3. Results

### 3.1. Nutrient digestibility

Compared to the control group, the apparent digestibility of total phosphorus in the basal diet was significantly increased (*P* < 0.05) in groups supplemented with 4.8 and 6.4 mg/kg QG (Table 3). In addition, the apparent digestibility of crude protein in 6.4 mg/kg QG supplement group was higher than that in 3.2 mg/kg QG supplement group (*P* < 0.05). However, there were no significant differences in apparent digestibility of crude fat, crude protein, and calcium in the experimental groups compared to the control group (*P* > 0.05).

**Table 3:** Effect of quercetagetin on apparent metabolic rate of nutrients in broilers (%).

**Items**	**Control group**	**The amount of quercetagetin**			**SEM**	***P*-value**
		**3.2 mg/kg**	**4.8 mg/kg**	**6.4 mg/kg**		
Ether extract	83.14 ± 1.57	82.65 ± 1.18	85.55 ± 2.32	85.57 ± 2.13	0.510	0.093
Crude protein	62.92 ± 3.53^a^^b^	62.30 ± 3.23^b^	65.43 ± 2.33^a^^b^	66.42 ± 2.17^a^	0.680	0.044
Calcium	54.70 ± 2.04	56.89 ± 1.67	56.11 ± 2.78	55.82 ± 2.00	0.466	0.481
Total phosphorus	52.62 ± 2.94^b^	53.57 ± 3.34^a^^b^	56.56 ± 2.86^a^	57.05 ± 2.30^a^	0.700	0.040

a, b Within a row, means with different superscripts differ significantly (*P* < 0.05). Values are means ± standard deviation (*n* = 10).

SEM, the standard error of the means.

### 3.2. Intestinal morphology

Compared to the control group, the villus height in the jejunum and ileum of broilers fed on a basal diet supplemented with 4.8 mg/kg QG was significantly increased, while the crypt depth in the jejunum and ileum in the three experimental groups was significantly decreased (*P* < 0.05, Table 4). However, the ratios of villus height to crypt depth in the jejunum and ileum of broilers in the experimental groups were significantly increased (*P* < 0.05) compared to the control group.

**Table 4:** Effect of quercetagetin on intestinal morphology of broilers.

**Items**	**Control group**	**The amount of quercetagetin**			**SEM**	***P*-value**
		**3.2 mg/kg**	**4.8 mg/kg**	**6.4 mg/kg**		
**Jejunum**						
Villus height, μm	1296.04 ± 123.11^b^	1317.50 ± 54.89^b^	1430.49 ± 201.22^a^	1385.10 ± 102.00^a^^b^	17.207	0.009
Crypt depth, μm	190.44 ± 52.75^a^	152.52 ± 26.56^b^	161.52 ± 42.85^b^	163.98 ± 27.17^b^	4.688	0.031
Villus height/crypt depth	7.30 ± 2.09^b^	8.87 ± 1.46^a^	9.34 ± 2.48^a^	8.64 ± 1.41^a^	0.240	0.009
**Ileum**						
Villus height, μm	1114.26 ± 140.30^b^	1172.57 ± 52.42^a^^b^	1200.79 ± 100.62^a^	1131.71 ± 115.84^a^^b^	11.871	0.046
Crypt depth, μm	203.22 ± 41.21^a^	150.59 ± 29.51^b^	153.20 ± 43.45^b^	155.09 ± 31.35^b^	4.703	< 0.001
Villus height/Crypt depth	5.76 ± 1.63^b^	8.08 ± 1.66^a^	8.36 ± 2.20^a^	7.56 ± 1.70^a^	0.222	< 0.001

a, b Within a row, means with different superscripts differ significantly (*P* < 0.05). Values are means ± standard deviation (*n* = 10).

SEM, the standard error of the means.

### 3.3. Immune-organ index

There were no significant differences in spleen, thymus, and bursa of fabricius indexes in the experimental groups compared to the control group (*P* > 0.05, Table 5).

**Table 5:** Effect of quercetagetin on organ indices of broilers (g/kg).

**Items**	**Control group**	**The amount of quercetagetin**			**SEM**	***P*-value**
		**3.2 mg/kg**	**4.8 mg/kg**	**6.4 mg/kg**		
Spleen	1.03 ± 0.14	1.06 ± 0.16	1.13 ± 0.03	1.24 ± 0.33	0.021	0.359
Thymus	3.35 ± 0.83	3.84 ± 0.37	3.83 ± 0.63	4.27 ± 0.64	0.178	0.307
Bursa	2.26 ± 0.66	2.50 ± 0.54	2.25 ± 0.37	2.48 ± 0.46	0.126	0.872

Values are means ± standard deviation (*n* = 10).

SEM, the standard error of the means.

### 3.4. Blood index

The T-AOC activity in broilers supplemented with 3.2 mg/kg QG in feed was significantly increased relative to the control. In addition, the C4 content in the blood of chickens supplemented with 3.2 and 4.8 mg/kg QG was significantly increased (*P* < 0.05, Table 6). At the same time, IgG level, GSH-Px, and SOD activities in the blood of all experimental groups were significantly increased (*P* < 0.05) compared to the control.

**Table 6:** Effect of quercetagetin on serum biochemical indices of broilers.

**Items**	**Control group**	**The amount of quercetagetin**			**SEM**	***P*-value**
		**3.2 mg/kg**	**4.8 mg/kg**	**6.4 mg/kg**		
IgA, μg/mL	515.01 ± 62.18	531.10 ± 10.58	573.23 ± 55.56	580.04 ± 11.39	14.250	0.342
IgG, μg/mL	817.01 ± 95.47^b^	1043.73 ± 140.43^a^	1138.92 ± 156.74^a^	1106.14 ± 42.25^a^	42.547	0.010
IgM, μg/mL	8.54 ± 0.41	10.48 ± 0.65	10.59 ± 1.11	10.36 ± 0.96	0.305	0.054
C3, g/L	2.59 ± 0.46	2.87 ± 0.45	2.87 ± 0.28	2.89 ± 0.31	0.101	0.552
C4, g/L	1.84 ± 0.20^b^	2.19 ± 0.15^a^	2.16 ± 0.18^a^	2.03 ± 0.09^a^^b^	0.057	0.001
T-AOC, U/mL	0.24 ± 0.03^b^	0.29 ± 0.03^a^	0.27 ± 0.01^a^^b^	0.25 ± 0.06^a^^b^	0.012	< 0.001
GSH-Px, U/mL	514.68 ± 39.31^b^	612.19 ± 67.24^a^	630.72 ± 71.09^a^	594.32 ± 54.98^a^	14.600	0.015
SOD, U/mL	149.17 ± 43.83^b^	218.31 ± 20.21^a^	214.87 ± 28.31^a^	221.44 ± 10.85^a^	9.990	0.009
MDA, nmol/mL	3.01 ± 0.22	2.76 ± 0.09	2.84 ± 0.42	2.82 ± 0.23	0.068	0.717

a, b Within a row, means with different superscripts differ significantly (*P* < 0.05). Values are means ± standard deviation (*n* = 10).

IgA, immunoglobulin A; IgG, immunoglobulin G; IgM, immunoglobulin M; C3, complement 3; C4, complement 4; T-AOC, total antioxidant capacity; GSH-Px, glutathione peroxidase; SOD, superoxide dismutase; MDA, malondialdehyde; SEM, the standard error of the means.

### 3.5. Antioxidant indexes of the jejunum and ileum mucosa

The T-AOC activity in the jejunum mucosa and the T-AOC and GSH-Px activities in the ileum mucosa of broilers supplemented with 4.8 mg/kg QG were significantly increased relative to the control (*P* < 0.05). In addition, the T-AOC activity in the ileum mucosa of chickens supplemented with 6.4 mg/kg QG was significantly increased compared to the control (*P* < 0.05, Table 7).

**Table 7:** Effect of quercetagetin on antioxidant performance of intestinal mucosa of broilers.

**Items**	**Control group**	**The amount of quercetagetin**			**SEM**	***P-*value**
		**3.2 mg/kg**	**4.8 mg/kg**	**6.4 mg/kg**		
**Jejunal mucosa**						
T-AOC, U/mg prot	0.61 ± 0.06^b^	0.62 ± 0.08^b^	0.70 ± 0.07^a^	0.66 ± 0.06^a^^b^	0.015	0.129
GSH-Px, U/mg prot	42.99 ± 8.97	49.4 ± 15.48	48.03 ± 10.57	42.25 ± 6.7	2.176	0.596
SOD, U/mg prot	570.43 ± 13.18	558.21 ± 14.55	566.75 ± 33.11	555.07 ± 14.14	4.948	0.326
MDA, nmol/mg prot	0.17 ± 0.03	0.22 ± 0.08	0.21 ± 0.03	0.20 ± 0.09	0.038	0.179
**Ileum mucosa**						
T-AOC, U/mg prot	0.60 ± 0.06^b^	0.62 ± 0.02^a^^b^	0.71 ± 0.08^a^	0.72 ± 0.10^a^	0.020	0.038
GSH-Px, U/mg prot	61.65 ± 8.78^b^	59.30 ± 13.73^b^	74.71 ± 7.11^a^	64.37 ± 10.75^a^^b^	2.327	0.044
SOD, U/mg prot	534.49 ± 12.99	538.47 ± 12.12	532.58 ± 22.38	538.51 ± 23.82	4.212	0.957
MDA, nmol/mg prot	0.12 ± 0.04	0.14 ± 0.04	0.15 ± 0.02	0.15 ± 0.05	0.010	0.741

a, b Within a row, means with different superscripts differ significantly (*P* < 0.05). Values are means ± standard deviation (*n* = 10).

T-AOC, total antioxidant capacity; GSH-Px, glutathione peroxidase; SOD, superoxide dismutase; MDA, malondialdehyde; SEM, the standard error of the means.

### 3.6. Antioxidant indexes of liver

The SOD activity in the liver of broilers supplemented with 6.4 mg/kg QG in feed was significantly increased relative to the control group (*P* < 0.05). Meanwhile, the MDA level in the liver of groups supplemented with 3.2 and 6.4 mg/kg QG was significantly decreased (*P* < 0.05, Table 8).

**Table 8:** Effect of quercetagetin on liver antioxidant performance of broilers.

**Items**	**Control group**	**The amount of quercetagetin**			**SEM**	***P*-value**
		**3.2 mg/kg**	**4.8 mg/kg**	**6.4 mg/kg**		
T-AOC, U/mg prot	0.83 ± 0.16	0.90 ± 0.12	0.91 ± 0.13	0.94 ± 0.12	0.027	0.648
GSH-Px, U/mg prot	120.16 ± 23.47	142.99 ± 35.9	153.73 ± 31.36	129.11 ± 18.53	5.989	0.206
SOD, U/mg prot	663.46 ± 46.78^b^	703.13 ± 51.06^a^^b^	727.59 ± 69.86^a^^b^	748.00 ± 73.93^a^	14.205	0.034
MDA, nmol/mg prot	1.31 ± 0.02^a^	1.19 ± 0.07^b^	1.36 ± 0.05^a^	1.17 ± 0.10^b^	0.026	0.012

a, b Within a row, means with different superscripts differ significantly (*P* < 0.05). Values are means ± standard deviation (*n* = 10).

T-AOC, total antioxidant capacity; GSH-Px, glutathione peroxidase; SOD, superoxide dismutase; MDA, malondialdehyde; SEM, the standard error of the means.

### 3.7. Expression of antioxidation-related genes in the intestinal mucosa

#### 3.7.1. Expression of antioxidation-related genes in jejunum mucosa

**Figure 1:**
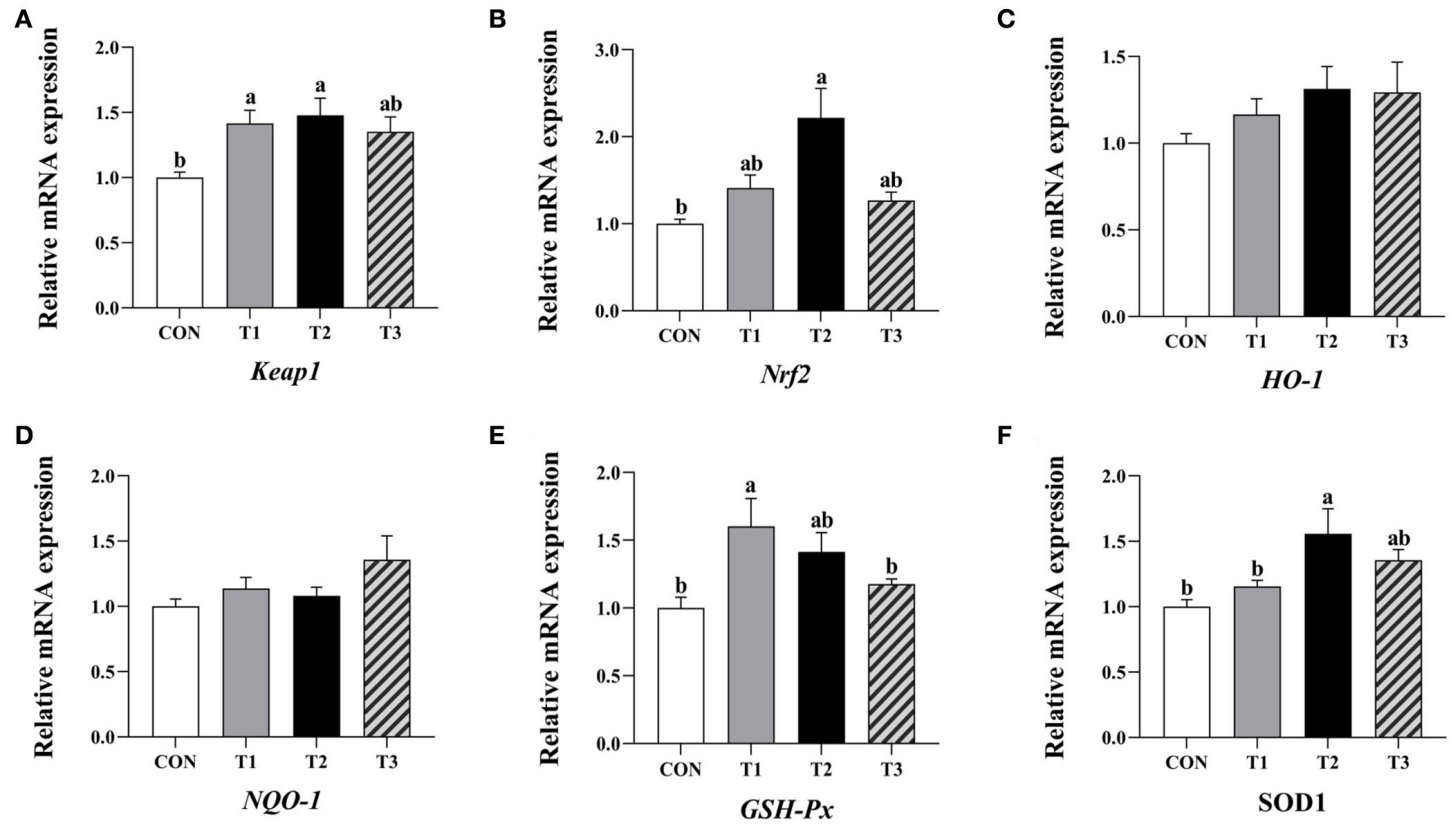
Effect of quercetagetin on expression level of antioxidant related genes in jejunal mucosa of broilers. Values are means ± standard deviation (*n* = 10). Above the bar no letter or the same letter mean no significant (*P* > 0.05), while with different letter mean significant difference (*P* < 0.05). Kelch-like ECH-associated protein 1 (Keap1) **(A)**, Nuclear factor E2 related factor 2 (Nrf2) **(B)**, Heme oxygenase-1 (HO-1) **(C)**, NAD(P)H: quinone oxidoreductase 1 (NQO-1) **(D)**, Glutathione peroxidase (GSH-Px) **(E)**, Superoxide dismutase 1 (SOD1) **(F)**.

Compared to the control group, the relative expression levels of *Keap1* and *GSH-Px* mRNA in the jejunum mucosa of broilers supplemented with 3.2 mg/kg QG and the *Keap1, Nrf2*, and *SOD1* mRNA levels in the group supplemented with 4.8 mg/kg QG was significantly increased (*P* < 0.05, Figure 1).

#### 3.7.2. Expression of antioxidation-related genes in the ileum

**Figure 2:**
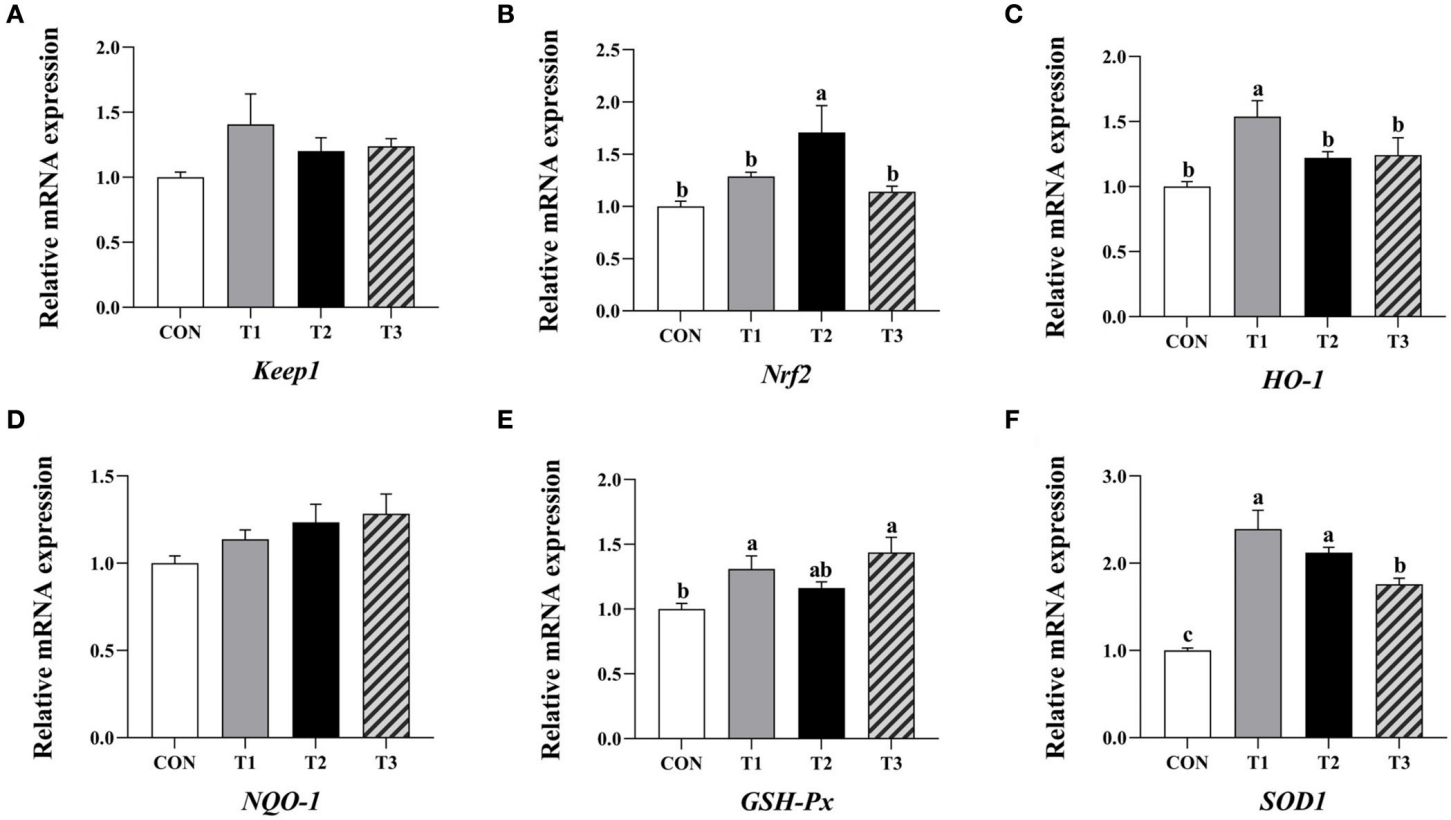
Effect of quercetagetin on expression level of antioxidant related genes in inileal mucosa of broilers. Values are means ± standard deviation (*n* = 10). Above the bar no letter or the same letter mean no significant (*P* > 0.05), while with different letter mean significant difference (*P* < 0.05). Kelch-like ECH-associated protein 1 (Keap1) **(A)**, Nuclear factor E2 related factor 2 (Nrf2) **(B)**, Heme oxygenase-1 (HO-1) **(C)**, NAD(P)H: quinone oxidoreductase 1 (NQO-1) **(D)**, Glutathione peroxidase (GSH-Px) **(E)**, Superoxide dismutase 1 (SOD1) **(F)**.

Compared to the control group, the relative expression levels of *HO-1, GSH-Px*, and *SOD1* mRNA in the ileum mucosa of broilers supplemented with 3.2 mg/kg QG, *Nrf2*, and *SOD1* mRNA in the group supplemented with 4.8 mg/kg QG, and *GSH-Px* and *SOD1* mRNA in the group supplemented with 6.4 mg/kg QG were significantly increased (*P* < 0.05, Figure 2).

### 3.8. Expression of antioxidation-related genes in the liver

**Figure 3:**
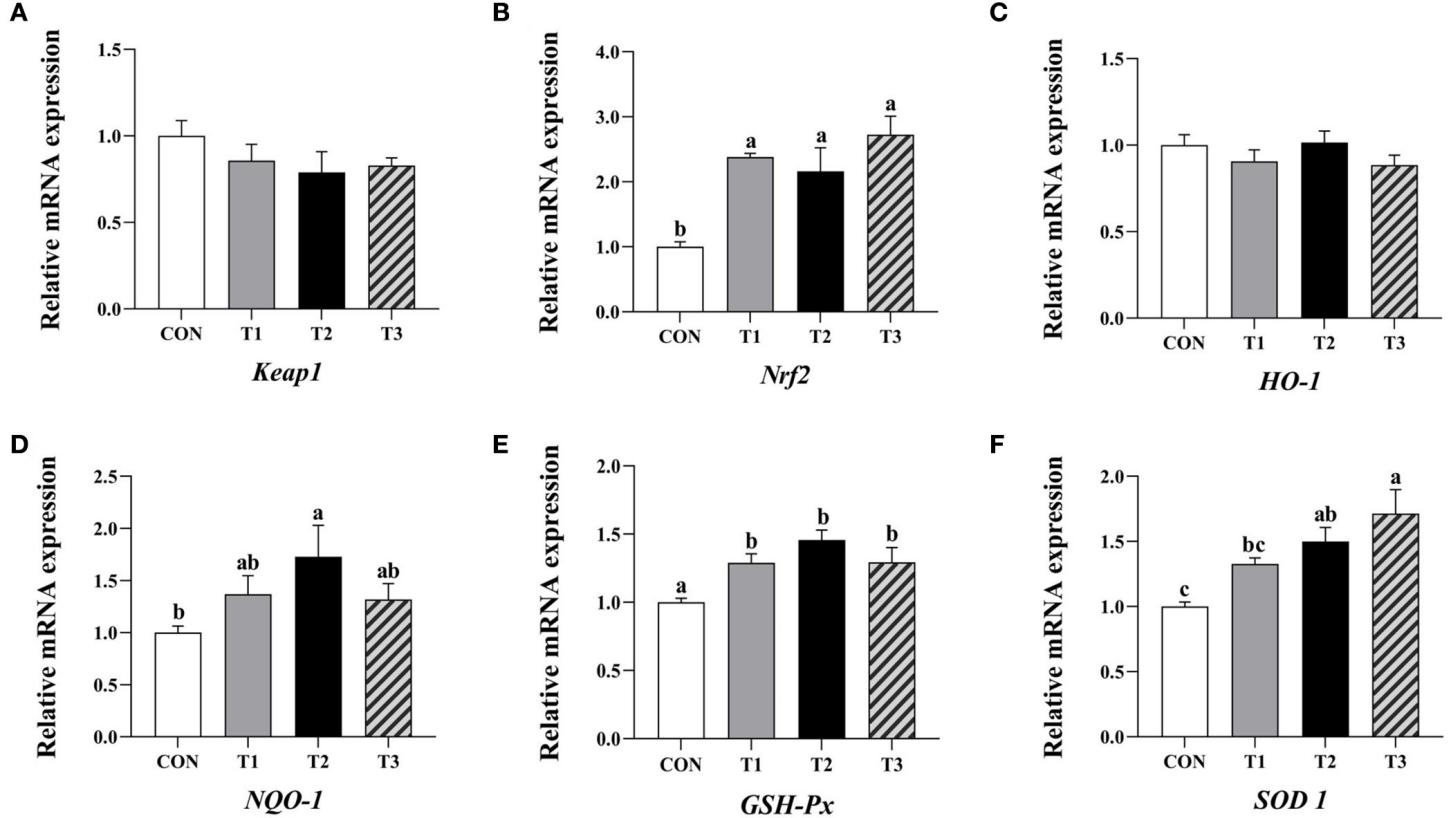
Effect of quercetagetin on expression level of antioxidant related genes in liver of broilers. Values are means ± standard deviation (*n* = 10). Above the bar no letter or the same letter mean no significant (*P* > 0.05), while with different letter mean significant difference (*P* < 0.05). Kelch-like ECH-associated protein 1 (Keap1) **(A)**, Nuclear factor E2 related factor 2 (Nrf2) **(B)**, Heme oxygenase-1 (HO-1) **(C)**, NAD(P)H: quinone oxidoreductase 1 (NQO-1) **(D)**, Glutathione peroxidase (GSH-Px) **(E)**, Superoxide dismutase 1 (SOD1) **(F)**.

The relative expression levels of *Nrf2* and *GSH-Px* mRNA in the liver of broilers in the three experimental groups were significantly increased compared to the control (*P* < 0.05, Figure 3). In addition, the relative expression levels of *NQO-1* and *SOD1* mRNA in the liver of broilers supplemented with 4.8 mg/kg QG in feed, and *SOD1* mRNA in the group supplemented with 6.4 mg/kg QG were significantly increased compared to the control (*P* < 0.05).

## 4. Discussion

Flavonols have numerous biological functions. For instance, quercetin enhances the antioxidant capacity of broilers' intestines, alleviates intestinal inflammatory reactions, regulates the internal intestinal environment, maintains the integrity of the intestinal barrier, and improves the structure and morphology of the intestinal tract (15–17). Rutin exhibits antioxidant and anti-inflammatory effects by inhibiting the production of reactive oxygen species and cell apoptosis (18). In the present study, the dose range of QG was selected based on the results of free radical scavenging experiments of QG *in vitro* and the pre-experiments of QG in broilers. And the results implied that the dietary supplementation with QG improved the morphology of broilers' jejunum and ileum, enhanced the functions. The positive effect of QG on the intestinal development of broilers is similar to that induced by quercetin, probably due to its antioxidant, anti-inflammatory, and antiviral activities, which strengthen intestinal resistance and homeostasis. Specifically, QG has a strong scavenging capacity against OH·, DPPH, and ABTS (12), significantly inhibits the intestinal inflammatory response induced by silver nanoparticles (AgNP) (19), and has a strong inhibitory effect against Chikungunya virus infection with IC_50_ of 13.85 μg/ml (43.52 μM) (20).

Flavonol compounds affect the utilization efficiency of some nutrients in broilers. For example, the relative expression levels of glucose transporter 2, peptide transporter 1, and fatty acid synthase mRNA in 1-day-old Ross 308 broilers fed with 200, 400, and 800 mg/kg quercetin for 35 d were significantly increased (17). Quercetin prevents the inhibitory effect of menadione and other glutathione-consuming substances on calcium absorption in broilers' intestines by alleviating oxidative stress and inhibiting the activation of the FasL/Fas/caspase-3 pathway (21). In addition, the results in this study revealed that dietary supplementation with QG has no significant effect on the apparent digestibility of crude protein, crude fat, and calcium in broilers but promotes the digestion and absorption of phosphorus (4.8 and 6.4 mg/kg QG supplement groups), which is key to reducing the feed cost. This is because dietary supplementation with QG improves the intestinal morphology of jejunum and ileum, promoting phosphorus absorption. In addition, QG supplements alleviate lipid and protein oxidation in the diet, improving the diet stability, which benefits nutrient absorption. Antioxidation induced by QG provides beneficial conditions for the absorption and utilization of nutrients. However, there were no significant differences in the apparent digestibility of crude protein, crude lipid, and calcium in this study, which may be related to the quantity of QG supplements fed to the broilers.

Flavonol compounds have immunomodulatory effects, with sufficient supplementation improving the immunity of broilers. For example, dietary supplementing Tianfu broilers with 0.4 g/kg quercetin for 14 weeks significantly increases the secretory immunoglobulin A concentrations in the duodenum, jejunum, and ileum (16). Besides, supplementing 1-day-old broilers with 0.5 mg/kg quercetin exerts immunomodulatory, antioxidant, and anti-apoptotic effects and alleviates ochratoxin A-induced immunotoxicity by regulating the PI3K/AKT pathway (22). At the same time, supplementing 1-day-old AA broilers with 0.02% quercetin for 6 weeks significantly increases the blood C3 content, while 0.06% quercetin significantly increases the thymus and spleen index, IgA, IgM, C4, interleukin 4, and tumor necrosis factor alpha contents in the blood (23). In the present study, dietary supplementation with QG improved the immunity of broilers by increasing IgG and C4 (3.2 and 4.8 mg/kg QG supplement groups) levels in the blood, given the antioxidant effect of QG, which alleviates oxidative stress on immune cells. On human lymphoblasts (Jurkat T), QG reduces the effects of oxidative stress by scavenging free radicals and enhancing antioxidant enzyme activity (24). Besides, the immunomodulatory and anti-inflammatory effects of QG influence the immune response of the body. Kang et al. (25) revealed that QG inhibits the formation of macrophage-derived chemokine (MDC/CCL22) in the human keratinocytes (HaCaT) by mediating signal transducer and activator of transcription 1 (STAT1), suppressor of cytokine signaling 1 (SOCS1) and transforming the growth factor-β1 (TGF-β1); hence its potential as an immunotherapeutic agent against inflammatory diseases such as atopic dermatitis (AD). QG also alleviates the effects of oxidative stress induced by silver nanoparticles on human neutrophils (19).

Flavonol compounds exhibit antioxidant activity; thus, sufficient supplements could improve the antioxidant capacity of broilers and alleviate the effects of oxidative stress and injury on broilers. Supplementation of a 1-day-old AA broilers diet with 400 and 800 mg/kg quercetin for 11 d significantly reduced MDA increase in the blood and intestinal mucosa induced by oxidized soybean oil. At the same time, the mRNA expression levels of *Nrf2, CAT, SOD1, GSH-Px2*, and *HO-1* in the AA broilers ileum mucosa were significantly up-regulated, the antioxidant capacity was improved, and the effect of oxidized soybean oil was alleviated (26). In addition, with supplementation of 1-day-old Ross 308 broilers diet with 1 g/kg rutin for 42 d, the SOD, CAT, and GSH-Px activities in the serum were significantly increased, but the MDA level was significantly decreased, which enhanced the antioxidant capacity of the broilers (27). In this study, the results revealed that QG enhanced the antioxidant capacity of the broilers through the Keap1-mediated Nrf2/ARE signaling pathway. Since Nrf2/ARE signaling pathway is a critical antioxidant and defense signaling pathway, the free radical scavenging capacity and antioxidant activity are promoted by regulating the expression of antioxidases, such as HO-1, NQO-1, GSH-Px, and SOD1. The antioxidant activity of quercetin correlates to the hydroxyl groups on three positions of the A and C rings and the catechol group on the B ring (28). Compared to quercetin, QG has an extra phenolic hydroxyl at six positions of the A ring. Furthermore, the oxygen atom on the 4-carbonyl group in the parent nucleus of QG has a stronger coordination ability, and the spatial structure of its polyhydroxy groups is conducive to the formation of metal complexes with various structures, which is an important source of antioxidant and other biological activities of QG (10). *In vitro*, QG has similar ABTS and DPPH scavenging activities to quercetin, with an IC_50_ of 12.16 ± 0.56 and 12.38 ± 0.50 μmol/L, and 27.12 ± 1.31 and 27.85 ± 1.13 μmol/L, respectively (29). Besides, major antioxidant components such as gallic acid, epigallocatechin, quercetin, and QG have been detected in an alcohol extract of defatted marigold residue, among which QG had the strongest antioxidant activity (30), which may be one of the reasons for the difference in the dosage of QG and quercetin used in broiler production. QG is extracted from marigold, which is rich in resources. The application of QG as feed additive in broiler production is economical and easily available.

## 5. Conclusions

Dietary supplementation with QG improves broilers' apparent digestibility of phosphorus in feed, improves the jejunum and ileum morphology, and enhances their immunity. In addition, QG increases the activity of antioxidant enzymes and strengthens the antioxidant capacity of broilers through the Keap1-mediated Nrf2/ARE signaling pathway.

## Data availability statement

The original contributions presented in the study are included in the article/supplementary material, further inquiries can be directed to the corresponding author/s.

## Ethics statement

The animal study was reviewed and approved by the Animal Care and Use Committee of Hebei Agriculture University (Baoding, China). All animal experiments complied with the ARRIVE guidelines and were carried out in accordance with the UK. Animals (Scientific Procedures) Act, 1986 and associated guide-lines, EU Directive 2010/63/EU for animal experiments.

## Author contributions

FW and HW are the primary investigators in this study. SL participated in the animal experiments. ZW participated in sample analysis and statistical data analysis. SH revised the manuscript. BC designed this study and wrote the manuscript as corresponding author. All authors read and approved the final manuscript.

## References

[1] Yan Y Chen XX Huang JP Huan CC Li CM. H_2_O_2_-induced oxidative stress impairs meat quality by inducing apoptosis and autophagy via ROS/NF-κB signaling pathway in broiler thigh muscle. Poult Sci. (2022) 101:101759. 10.1016/j.psj.2022.101759 35240354 PMC8889410

[2] Hafez MH El-Kazaz SE Alharthi B Ghamry HI Alshehri MA Sayed S. The impact of curcumin on growth performance, growth-related gene expression, oxidative stress, and immunological biomarkers in broiler chickens at different stocking densities. Animals. (2022) 12:958. 10.3390/ani12080958 35454205 PMC9024619

[3] Brannan KE Helfrich KK Flentke GR Smith SM Livingston KA Jansen van Rensburg C. Influence of incubation, diet, and sex on avian uncoupling protein expression and oxidative stress in market age broilers following exposure to acute heat stress. Poult Sci. (2022) 101:101748. 10.1016/j.psj.2022.101748 35278756 PMC8917286

[4] Liu LL Zhao LY Liu Y Yu XL Qiao XY. Rutin ameliorates cadmium-induced necroptosis in the chicken liver via inhibiting oxidative stress and MAPK/NF-κB pathway. Biol Trace Elem Res. (2022) 200:1799–810. 10.1007/s12011-021-02764-5 34091842

[5] Guo J Yan WR Tang JK Jin X Xue HH Wang T. Dietary phillygenin supplementation ameliorates aflatoxin B1-induced oxidative stress, inflammation, and apoptosis in chicken liver. Ecotoxicol Environ Saf . (2022) 236:113481. 10.1016/j.ecoenv.2022.113481 35405527

[6] Ahmadipour B Pat S Abaszadeh S Hassanpour H Khajali F. Pomegranate peel as a phytogenic in broiler chickens: influence upon antioxidant, lipogenesis and hypotensive response. Vet Med Sci. (2021) 7:1907–13. 10.1002/vms3.556 34132060 PMC8464295

[7] Abbas AO Alaqil AA El-Beltagi HS Abd El-Atty HK Kamel NN. Modulating laying hens productivity and immune performance in response to oxidative stress induced by E. Coli challenge using dietary propolis supplementation. Antioxidants. (2020) 9:893. 10.3390/antiox9090893 32967097 PMC7555396

[8] Li B Li W Tian Y Guo S Qian L Xu D. Selenium-alleviated hepatocyte necrosis and DNA damage in cyclophosphamide-treated geese by mitigating oxidative stress. Biol Trace Elem Res. (2020) 193:508–16. 10.1007/s12011-019-01717-3 31025241

[9] Gou Z Fan Q Li L Wang Y Lin X Cui X. High dietary copper induces oxidative stress and leads to decreased egg quality and reproductive performance of Chinese Yellow broiler breeder hens. Poult Sci. (2021) 100:100779. 10.1016/j.psj.2020.10.033 33518335 PMC7936131

[10] Xu LW Wang GY Shi YP. Chemical constituents from *Tagetes erecta* flowers. Chem Nat Compd. (2011) 47:281–3. 10.1007/s10600-011-9905-5

[11] Huang XQ Gao W Yun X Qing ZX Zeng JG. Effect of natural antioxidants from marigolds (*Tagetes erecta* L.) on the oxidative stability of soybean oil. Molecules. (2022) 27:2865. 10.3390/molecules27092865 35566214 PMC9105600

[12] Fuentes J de Camargo AC Atala E Gotteland M Olea-Azar C Speisky H. QUercetin oxidation metabolite present in onion peel protects Caco-2 cells against the oxidative stress, NF-kB activation, and loss of epithelial barrier function induced by NSAIDs. J Agric Food Chem. (2021) 69:2157–67. 10.1021/acs.jafc.0c07085 33591188

[13] NRC. Nutrient Requirements of Poultry. 9th ed. Washington, DC: National Academies Press (1994).

[14] AOAC. Official Methods of Analysis. 18th ed. Arlington, VA: AOAC Int. (2006).

[15] Sun L Guo L Xu G Li Z Appiah MO Yang L. Quercetin reduces inflammation and protects gut microbiota in broilers. Molecules. (2022) 27:3269. 10.3390/molecules27103269 35630745 PMC9147699

[16] Amevor FK Cui Z Du X Ning Z Deng X Xu D. Supplementation of dietary quercetin and vitamin E promotes the intestinal structure and immune barrier integrity in aged breeder hens. Front Immunol. (2022) 13:860889. 10.3389/fimmu.2022.860889 35386687 PMC8977514

[17] Abdel-Latif MA Elbestawy AR El-Far AH Noreldin AE Emam M Baty RS. Quercetin dietary supplementation advances growth performance, gut microbiota, and intestinal mrna expression genes in broiler chickens. Animals. (2021) 11:2302. 10.3390/ani11082302 34438756 PMC8388376

[18] Rana AK Sharma S Saini SK Singh D. Rutin protects hemorrhagic stroke development via supressing oxidative stress and inflammatory events in a zebrafish model. Eur J Pharmacol. (2022) 925:174973. 10.1016/j.ejphar.2022.174973 35469838

[19] Rufino AT Ramalho A Sousa A de Oliveira JMPF Freitas P Gómez MAG. Protective role of flavonoids against intestinal pro-inflammatory effects of silver nanoparticles. Molecules. (2021) 26:6610. 10.3390/molecules26216610 34771019 PMC8588041

[20] Seyedi SS Shukri M Hassandarvish P Oo A Shankar EM Abubakar S. Corrigendum: computational approach towards exploring potential anti-chikungunya activity of selected flavonoids. Sci Rep. (2016) 6:26368. 10.1038/srep26368 27240753 PMC4886183

[21] Marchionatti AM Pacciaroni A Tolosa de Talamoni NG. Effects of quercetin and menadione on intestinal calcium absorption and the underlying mechanisms. Comp Biochem Physiol Mol Integr Physiol. (2013) 164:215–20. 10.1016/j.cbpa.2012.09.007 23000882

[22] Abdelrahman RE Khalaf AAA Elhady MA Ibrahim MA Hassanen EI Noshy PA. Quercetin ameliorates ochratoxin a-induced immunotoxicity in broiler chickens by modulation of PI3K/AKT pathway. Chem Biol Interact. (2022) 351:109720. 10.1016/j.cbi.2021.109720 34717913

[23] Yang JX Maria TC Zhou B Xiao FL Wang M Mao YJ. Quercetin improves immune function in Arbor Acre broilers through activation of NF-κB signaling pathway. Poult Sci. (2020) 99:906–13. 10.1016/j.psj.2019.12.021 32029167 PMC7587811

[24] Chkhikvishvili I Sanikidze T Gogia N Enukidze M Machavariani M Kipiani N. Constituents of French marigold (*Tagetes patula* L.) flowers protect jurkat T-cells against oxidative stress. Oxid Med Cell Longev. (2016) 2016:4216285. 10.1155/2016/4216285 27433287 PMC4940552

[25] Kang GJ Han SC Kang NJ Koo DH Park DB Eun SY. Quercetagetin inhibits macrophage-derived chemokine in HaCaT human keratinocytes via the regulation of signal transducer and activator of transcription 1, suppressor of cytokine signalling 1 and transforming growth factor-β1. Br J Dermatol. (2014) 171:512–23. 10.1111/bjd.12938 24602010

[26] Dong Y Lei J Zhang B. Effects of dietary quercetin on the antioxidative status and cecal microbiota in broiler chickens fed with oxidized oil. Poult Sci. (2020) 99:4892–903. 10.1016/j.psj.2020.06.028 32988526 PMC7598137

[27] Hassan FAM Roushdy EM Kishawy ATY Zaglool AW Tukur HA Saadeldin IM. Growth performance, antioxidant capacity, lipid-related transcript expression and the economics of broiler chickens fed different levels of rutin. Animals. (2018) 9:7. 10.3390/ani9010007 30583506 PMC6357029

[28] Boots AW Haenen GR Bast A. Health effects of quercetin: from antioxidant to nutraceutical. Eur J Pharmacol. (2008) 585:325–37. 10.1016/j.ejphar.2008.03.008 18417116

[29] Wang WY Xu HG Chen H Tai KD Liu FG Gao YX. *In vitro* antioxidant, anti-diabetic and antilipemic potentials of quercetagetin extracted from marigold (*Tagetes erecta* L.) inflorescence residues. J Food Sci Technol. (2016) 53:2614–24. 10.1007/s13197-016-2228-6 27478217 PMC4951414

[30] Gong Y Liu X He WH Xu HG Yuan F Gao YX. Investigation into the antioxidant activity and chemical composition of alcoholic extracts from defatted marigold (*Tagetes erecta* L.) residue. Fitoterapia. (2012) 83:481–9. 10.1016/j.fitote.2011.12.013 22223143

